# Mutation screening and genotype phenotype correlation of α-crystallin, γ-crystallin and GJA8 gene in congenital cataract

**Published:** 2011-03-11

**Authors:** Manoj Kumar, Tushar Agarwal, Sudarshan Khokhar, Manoj Kumar, Punit Kaur, Tara Sankar Roy, Rima Dada

**Affiliations:** 1 Laboratory for Molecular Reproduction and Genetics, Department of Anatomy, All India Institute of Medical Sciences, New Delhi, India; 2 Dr. Rajendra Prasad Centre for Ophthalmic Sciences, All India Institute of Medical Sciences, New Delhi, India; 3 Department of Biophysics, All India Institute of Medical Sciences, New Delhi, India; 4 Department of Anatomy, All India Institute of Medical Sciences, New Delhi, India

## Abstract

**Purpose:**

To screen α-crystallin (*CRYAB*), γ-crystallin (*CRYGC* and *CRYGD*), and Connexin 50 (Cx-50 or *GJA8*) genes in congenital cataract patients and controls.

**Methods:**

Thirty clinically diagnosed congenital cataract cases below 3 years of age from northern India, presenting at Dr. R. P. Centre for Ophthalmic Sciences (AIIMS, New Delhi, India) were enrolled in this study. Genomic DNA was extracted from peripheral blood, all coding and exon/intron regions were amplified using PCR and direct sequencing was performed to detect any nucleotide variation. ProtScale and Discovery Studio programs were used for insilico and structural analysis of non-synonymous mutations.

**Results:**

DNA sequencing analysis of *CRYAB, CRYGC, CRYGD*, and *GJA8* showed a total of six variations of which two were novel (*CRYGC*:p.R48H and *GJA8*:p.L281C) and four have been previously reported (*CRYAB*: rs11603779T>G, *GJA8*: p.L268L, *CRYGD*: p.R95R, and c.T564C). Both the novel changes, in *CRYGC* and *GJA8* were found in 16.6% of the patients. Previously reported nucleotide alterations (*CRYGD*:p.R95R and c.T564C) were found in 90% of the patients. Insilico and structural analysis data suggested that two novel non-synonymous mutations altered the stability and solvent accessibility of γC-crystallin and Cx-50 proteins which may lead to lens opacification.

**Conclusions:**

We observed two novel nonsynonymous variations and four reported variations in *CRYAB, CRYGC, CRYGD*, and *GJA8*. The p.R48H variation in γC-crystallin may disrupt the normal structure of lens and can cause cataract. Cx50 is responsible for joining the lens cells into a functional syncytium and a mutation (p.L281C) in *GJA8* may lead to lens opacification resulting in cataract formation. This study further expands the mutation spectrum of congenital cataract and help understanding how mutant proteins lead to opacification of lens.

## Introduction

Cataract is an opacification of the lens resulting from alterations in lens cellular architecture or in lens proteins, or both. Congenital cataract is a clinically and genetically heterogeneous lens disease responsible for a significant proportion of visual impairment and blindness in childhood [1,2]. The prevalence of congenital cataract is estimated to vary from 0.6 to 6 per 10,000 live births with an incidence of 2.2–2.49 per 10,000 live births [3]. It is estimated that globally, 20 million children under the age of 16 years suffer from cataract, and among these, 200,000 (15%) are severely visually impaired or blind [4,5]. Pediatric cataracts are responsible for 7.4% to 15.3% of childhood blindness in developing countries like india [6-8]. The prevalence of blindness in children ranges from approximately 0.3/1000 children in affluent regions to 1.5/1000 in the poorest communities. The population of India in 2001 was estimated to be 1.03 billion, approximately 420 million of whom are children under 16 years of age (40.9%). Overall, there are probably 280,000–320,000 blind children in India [9]. Cataract is responsible for ~12% of childhood blindness in India [6,10]. The occurrence of congenital cataract varies in different parts of India as it is 5.25% in northen India [7], 8.5% in northeast states [6], 7.25% in western India [11] and 11.4% in south India [12].

Currently there are ~60 loci implicated in non-syndromic congenital cataract, among these, over 22 have been associated with mutations in specific genes [13-15] including 10 crystallin genes: αA-crystallin (*CRYAA*), αB-crystallin (*CRYAB*), βA1-crystallin (*CRYBA1*), βA4-crystallin (*CRYBA4*), βB1-crystallin (*CRYBB1*), βB2-crystallin (*CRYBB2*), βB3-crystallin (*CRYBB3*), γC-crystallin (*CRYGC*), γD-crystallin (*CRYGD*), γS-crystallin (*CRYGS*) [16-21], two cytoskeletal protein genes: beaded filament structural protein 1, filensin (*BFSP1*), beaded filament structural protein 2, phakinin (*BFSP2*) [22], four membrane protein genes: gap junction protein (alpha 3, *GJA3*), gap junction protein (alpha 8, *GJA8*), major intrinsic protein of lens fiber (*MIP*), and lens intrinsic membrane protein 2 gene (*LIM2*) [23-25], three growth and transcription factor genes: heat shock transcription factor 4 (*HSF4*), paired-like homeodomain 3 (*PITX3*), Maf-like protein (*MAF*) [26,27], and chromatin modifying protein-4B (*CHMP4B*), Ephrin receptor EphA2 (*EPHA2*), and Nance-Horan syndrome (*NHS*) [27].

As crystallin genes account for nearly 90% of the water soluble proteins in lens and the encoded proteins account for around 30% of lens mass, these proteins play an essential roles in maintaining the lens transparency [28] and are good candidate genes for screening in congenital cataract patients. Crystallin mutations accounts for about 50% of the non syndromic cataract [14]. Since the lens is an avascular structure, the crystallins are retained in soluble form through the maintenance of ionic balance by the actions of gap junction proteins which allow the metabolically active epithelium to regulate the precise inter-cellular communication and transport between the lens periphery and its interior. Gap junction proteins (alpha 3 and alpha 8) are expressed in the lens vesicle and mutations in these genes have been reported to lead to cataract [29-31].

Congenital cataract is the most important treatable cause of pediatric blindness in developing countries like India. In this pilot study, we screened 30 cases of congenital cataract for sequence variations in *CRYAB*, *CRYGC*, *CRYGD*, and *GJA8*. Upon sequence analysis we detected six sequence variations. Out of six, two novel mutations were found in *CRYGC* (R48H) and *GJA8* (L281C). The probable pathogenicity of the mutations found in this study as disease causing is discussed in light of earlier studies.

## Methods

### Clinical examination and selection of cases

After receiving ethical approval from the institutional review board (IRB#00006862; All India Institute of Medical Sciences, Delhi, India), 30 clinically diagnosed consecutive congenital cataract cases below 3 years of age from northern India, presenting at the Dr. R. P. Centre for Ophthalmic Sciences (AIIMS, New Delhi, India) were enrolled in this study. These congenital cataract cases had no other ocular or systemic abnormalities. Detailed history was taken from parents regarding high fever, TORCHES ([*Toxoplasma gondii*; *T*. *gondii*], rubella virus [RV], cytomegalovirus [CMV], herpes simplex virus [HSV], syphilis [caused by *Treponema pallidum*]) infection, tuberculosis, exposure to radiation, and drug intake during gestation period. Metabolic tests like serum biochemistry for levels of blood glucose, calcium and phosphorous evaluations, RBC transferase and galactokinase levels and urine test for reducing sugars (galactosemia) and for amino acids (Lowe syndrome) were also done. Cases with known cause of congenital cataract were excluded from the study. Affected status was determined by a history of cataract extraction or ophthalmologic examination. A total of 30 ethnically and age-matched normal individuals without any history of ocular or systemic disorders were enrolled as controls. They had no metabolic, genetic, or ocular disorder on examination by a ophthalmologist and an extensive history was taken regarding family, occupation of parents, any medical problem, and drug intake by parents. Informed consent in accordance with the Declaration of Helsinki was obtained from all participants or their parents and controls.

### DNA isolation, PCR amplification and sequence analysis

Genomic DNA was extracted from whole blood samples of all cases and controls, using organic method as described by Sambrook et al. [32] with some modifications. Briefly, 5 ml blood was incubated in 15 ml of Red Cell Lysis Buffer (RCLB) at 4 °C and then centrifuged at 6,861× g for 15 min at 4 °C. Supernatant was discarded and pellet was given repeated washes with RCLB till the pellet became white. The white pellet was re-suspended in 5 ml of DNA extraction buffer, 40 µl of proteinase-K (40 µg/ml) and 300 µl of sodium dodecyl sulphate (SDS). The cocktail was incubated at 55 °C for 2-3 h. The digested proteins were precipitated by adding equal volumes of saturated phenol and chloroform:isoamylalcohol (24:1). The mixture was gently mixed on rotor-mixer for 15-20 min and then centrifuge at 6,861× g for 15 min at 4 °C. In upper viscous layer equal amount of chloroform:isoamylalcohol (24:1) solution was added and again mixed for 15 min in rotor-mixer. The aqueous layer containing the genomic DNA was carefully collected. Then DNA was precipitated with chilled ethanol and centrifugation at 1,461.6× g for 15 min. DNA pellet was washed with 70% ethanol and dissolved in TE buffer. The exon-intron regions of *CRYAB*, *CRYGC*, *CRYGD*, and *GJA8* were amplified in congenital cataract patients and controls. PCR amplifications for all primer sets (Table 1) were performed in a 40 μl volume containing 1.0 μl of 20 mM stock solution for each primer (Eurofins Genomics India pvt Ltd, Bangalore, India), 100 ng of genomic DNA, 1 unit of Taq polymerase (Banglore Genei, Bengaluru, Karnataka, India), 0.1 mM of each deoxynucleotide triphosphate (dNTP), and 4 μl of 10× PCR buffer (with 15 mM MgCl_2_). Amplified PCR products were purified using a gel/PCR DNA fragments extraction kit (Geneaid Biotech Ltd., Sijhih City, Taiwan). Purified PCR products were sent for sequencing to Molecular Cloning Laboratories (South San Francisco, CA). All fragments were sequenced in both forward and reverse directions for confirmation of any nucleotide variation in congenital cataract patients and controls and compared to the Human Genome Reference Sequence (NC_000002.11 and NC_000001.10) provided by the National Center for Biotechnology Information (NCBI), using ClustalW2 (multiple sequence alignment program for DNA; European Bioinformatics Institute, Wellcome Trust Genome Campus, Hinxton, Cambridge, UK).

**Table 1:** Oligonucleotides used as primers for PCR amplification of *CRYAB*, *CRYGC*, *CRYGD*, and *GJA8* and their annealing temperatures.

**Gene (exon)**	**Forward primer (5′→3′)**	**Reverse primer (5′→3′)**	**Temperature (°C)**
*CRYAB* (1)	CCTGACATCACCATTCCAGA	GGCAGGGTAGGAAAGGAAA	51
*CRYAB* (2)	TGCAGAATAAGACAGCACCTG	CCAGCCTCCAAAGCTGATAG	54
*CRYAB* (3)	TGTTGTCATGGCATTTGGTC	TCATTCACTGGTGGGGAAA	57
*CRYGC* (1,2)	CAGCCATCCTGCTATATAG	GGCATGATGGAAATCTAG	50
*CRYGC* (3)	GTTGGACAAATTCTGGAA	GCACAATGAAAGAATGAC	45
*CRYGD* (1)	AGAACACGAAAATGCCCTTG	GTCTCACAGGCCTGCTCCT	55
*CRYGD* (2)	GAGCTTCCTCCATCGC	CCTGGGTCCTGACTTGA	48
*CRYGD* (3)	GCTGGACTGCCTAACAATGC	CACATCTTGGTTGCCATTTG	55
*GJA8* (1)	TATGGGCGACTGGAGTTTCCT	CTCCATGCGGACGTAGTGCAC	65
*GJA8* (2)	CTCTGGGTGCTGCAGATCATC	CACAGAGGCCACAGACAACAT	55
*GJA8* (3)	CACTACTTCCTGTACGGGTTC	CTCTTGGTAGCCCCGGGACAA	60
*GJA8* (4)	GTCTCCTCCATCCAGAAAGCC	TCATACGGTTAGATCGTCTGA	58

### Computational assessment of missense mutations

We used an evolutionary model to predict the functional consequence of genetic variation in the ATP-binding cassette, sub-family A (*ABC1*), member 1 gene and tested these predictions through in vitro assessments of protein function [33]. We predicted the functional consequence of each variant using PANTHER. The probability that a given coding variant will cause a deleterious functional change is estimated by the substitution position-specific evolutionary conservation (sub-PSEC) score. SIFT (Sorting Intolerant From Tolerant) analysis tool was also used to predict the functional impact of missense changes identified in this study. SIFT is a sequence homology based tool that sorts intolerant from tolerant amino acid substitutions and predicts whether an amino acid substitution in a protein will have a phenotypic effect [34]. SIFT is based on the premise that protein evolution is correlated with protein function. Positions with normalized probabilities less than 0.05 are predicted to be deleterious and, those greater than or equal to 0.05 are predicted to be tolerated in case of SIFT. We have also used an improved splice site predictor tool [35] to predict whether a nucleotide change is likely to create a splice site.

### Protein modeling

The normal and mutant proteins were analyzed for their structure. Prediction of structure differences between wild and mutant were performed using Discovery Studio (DS) 2.0 (Accelrys Inc., San Diego, CA) [36]. The first step in homology modeling method was to find suitable homologus structure (template). Comparative modeling for GJA8 was not possible as the homology model has only 21% sequence identity whereas homology model for human γC-crystallin has 84% sequence identity, thus the comparative modeling for human γC-crystallin was possible using homology model.

### Comparative modeling of human γC-crystallin

The best available template for the modeling of 3-D structure of the human γC-crystallin was a high resolution (1.9 Å) crystal structure of mouse γC-crystallin (PDB ID=2V2U) [37]. The sequence identity and similarity between human and mouse γC-crystallin was found to be 84% and 91%, respectively. This template was used to build the homology model of human γC-crystallin using MODELER 9.2 program [38,39] available in Discovery Studio (DS) 2.0 (Accelrys Inc., San Diego, CA), a software package for molecular modeling and simulation. The model with the lowest energy among all the generated models was taken and its stereochemistry checked using the Ramachandran plot. The native model was solvated and further minimized using the available molecular dynamics (MD) simulation protocols to ensure the stability of the generated model. The 3-D model structure of human γC-crystallin mutant (Arg48His) was developed taking the model structure of wild type human γC-crystallin by using the “Build Mutant” protocol and altering the corresponding residue from Arg to His. The built model of mutant was optimized similar to the wild-type human γC-crystallin. The explicit solvent MD simulation was also performed similar to the wild type human γC-crystallin.

### Statistical analysis

The correlation coefficient between mutations in crystallin and gap junction protein genes and parameters like degree of opacification, morphology of congenital cataract, and visual acuity were calculated by spearman's test. p-value less than 0.05 is considered as significant. Statistical analyses were performed using graphpad software (GraphPad Software, Inc., La Jolla, CA).

## Results

### Clinical findings

**Figure 1:**
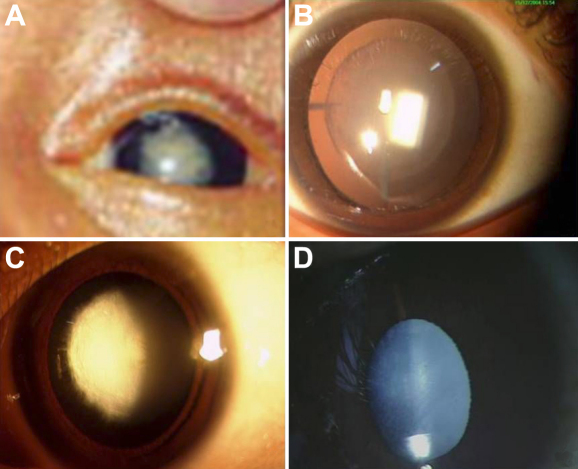
Different types of cataract. **A**: Nuclear cataract: The eye picture shows opacity of both lenses in one of the patient. **B**: Zonular/lamellar cataract: The zonular/lamellar cataract in one of the patient. **C**: Polar Cataract: Anterior polar cataract phenotype shown by one of the patient. **D**: Total Cataract: total cataract phenotype found in one patient.

A total of 30 congenital cataract patients below 3 years of age were enrolled in this study. The mean age of the patients was 1.75±0.19 years (one month to 3 years). The age of onset was recorded as the age at which the disease was first noticed by the parents or first documented by a clinician. All cases were sporadic and were enrolled consecutively as they presented to Dr. R.P. Centre for Ophthalmic Sciences. In this study 20 cases were males and 10 were females. None of the cases were product of consanguineous marriage and all cases had bilateral congenital cataract. The cataract phenotype varied among patients as 66.66% (20/30) of patients had nuclear cataract (Figure 1A), 23.33% (7/30) had zonular/lamellar type cataract (Figure 1B), 6.66% (2/30) had anterior polar cataract (Figure 1C), and 3.3% (1/30) had total cataract (Figure 1D; Table 2). In this study 93% cases were detected with one or the other nucleotide alterations in *CRYAB, CRYGC*, *CRYGD*, and *GJA8.* Six nucleotide variations were detected in patients (Table 3). 66% nucleotide changes were found in crystallin genes (*CRYAB*, *CYRGC*, and *CRYGD*) and 44% were detected in connexin (*GJA8*). Of the six mutations identified, two were novel and four have been reported.

**Table 2:** Mutations detected and the associated phenotype seen in patients. (Abbrevations: OD-Right eye, OS-Left eye, N/A-Not applicable, mm-millimeter).

					**Axial length (mm)**			
**Case number**	**Age/sex (years)**	**Age of onset (years)**	**Cataract phenotype**	**Corneal diameter (mm)**	**OD**	**OS**	**Visual acuity (decimal) binocular**	**Mutation detected**
CC1	0.9/F	since birth	pulverulent Nuclear cataract	11.0	19.00	19.40	N/A	CRYGC:p.R48H, CRYGD:p.R95R, CRYGD: c.T564C
CC2	3/M	9 months	Dense Nuclear cataract	11.2	20.10	20.50	0.20	CRYGD:p.R95R, CRYGD: c.T564C
CC3	3/M	1 year	Lamellar cataract with nystagmus	11.3	21.30	20.80	0.25	CRYGD:p.R95R, CRYGD: c.T564C
CC4	2.5/M	7 months	Nuclear cataract	11.8	20.50	20.90	0.17	CRYGD:p.R95R, CRYGD: c.T564C
CC5	3/M	1.2 years	Lamellar pulverulent	10.9	22.00	21.40	0.25	CRYGD:p.R95R
CC6	0.7/M	since birth	Nuclear cataract	10.0	19.30	19.80	N/A	CRYGD:p.R95R, CRYGD: c.T564C
CC7	3/M	8 months	Progressive lamellar cataract	11.0	21.30	20.70	0.20	CRYGD:p.R95R, CRYAB:rs11603779T>G
CC8	2.5/M	10 months	Nuclear cataract	11.2	20.70	21.00	0.20	CRYAB:rs11603779T>G
CC9	3/F	6 months	pulverulent Nuclear cataract	11.5	21.50	22.00	0.25	CRYGD:p.R95R, CRYGD: c.T564C
CC10	0.9/M	since birth	Nuclear cataract	11.1	20.00	19.60	N/A	CRYGD:p.R95R, CRYGD: c.T564C
CC11	3/F	2 months	Nuclear cataract	11.5	21.10	20.80	0.22	CRYGD:p.R95R, CRYGD: c.T564C
CC12	1.5/F	since birth	Anterior polar cataract	11.3	20.60	20.00	0.20	GJA8:p.L268L, CRYGD:p.R95R, CRYGD: c.T564C
CC13	0.6/M	since birth	Lamellar cataract with nystagmus	10.5	19.50	19.10	N/A	CRYGD:p.R95R, CRYGD: c.T564C
CC14	3/M	5 months	Nuclear cataract	11.6	21.60	21.10	0.25	CRYGD:p.R95R, CRYGD: c.T564C
CC15	3/M	7 months	Dense Nuclear cataract	11.8	22.10	22.30	0.22	CRYGD:p.R95R, CRYAB:rs11603779T>G
CC16	1.4/M	since birth	Nuclear cataract	11.3	21.10	21.30	0.20	CRYGD:p.R95R, CRYGD: c.T564C
CC17	1/M	since birth	Nuclear cataract	11.6	20.30	20.10	N/A	CRYGD:p.R95R, CRYGD: c.T564C
CC18	3/M	1.4 years	Nuclear cataract with nystagmus	11.7	21.80	22.10	0.20	CRYGC:p.R48H, CRYGD:p.R95R, CRYGD: c.T564C
CC19	3/M	4 months	Lamellar cataract	12.0	21.60	21.10	0.25	CRYGC:p.R48H, CRYGD: c.T564C
CC20	0.7/M	since birth	Lamellar cataract with nystagmus	11.2	19.20	19.30	N/A	CRYGC:p.R48H, GJA8: p.L281C, CRYGD:p.R95R, CRYGD: c.T564C
CC21	0.1/F	since birth	Nuclear cataract	09.0	18.10	18.40	N/A	CRYGD:p.R95R, CRYGD: c.T564C
CC22	0.3/M	since birth	Nuclear cataract	10.3	19.10	18.90	N/A	CRYAB:rs11603779T>G
CC23	1.2/F	2 months	pulverulent Nuclear cataract	11.4	20.50	20.40	0.25	CRYGD:p.R95R, CRYGD: c.T564C
CC24	0.9/M	since birth	Nuclear cataract	10.3	20.10	20.30	N/A	CRYGD:p.R95R
CC25	1/M	since birth	Anterior polar cataract	12.0	19.80	20.20	N/A	CRYGD:p.R95R
CC26	1.3/F	4 months	Nuclear cataract	11.6	21.30	21.10	0.25	CRYGD:p.R95R, CRYGD: c.T564C
CC27	1/F	since birth	Lamellar cataract	11.7	20.40	20.30	N/A	CRYGD:p.R95R, CRYGD: c.T564C
CC28	0.11/M	since birth	Nuclear cataract pulverulent	09.4	19.40	19.30	N/A	CRYGD:p.R95R, CRYGD: c.T564C
CC29	1.1/F	since birth	Total cataract	10.0	21.30	20.90	0.25	CRYGD:p.R95R, CRYGD: c.T564C
CC30	3/F	1 year	Nuclear cataract with nystagmus	11.0	22.10	22.50	0.20	CRYGD:p.R95R, CRYGD: c.T564C

**Table 3:** Nucleotide variations found in congenital Cataract patients.

**Sample number**	**Nucleotide change**	**Locus**	**Codon change**	**Amino acid change**	**Type of mutation**	**PANTHER/SIFT score**	**Frequency**
1	rs11603779T>G^	CRYAB	intronic	N/A	N/A	N/A	13.33%
2	c.G181A*	CRYGC	CGT>CAT	R48H	NS	−2.72/1.00	13.33%
3	c.T564C^	CRYGD	3′UTR	N/A	N/A	N/A	76.66%
4	c.A313G^	CRYGD	AGA>AGG	R95R	SYN	N/A	93.33%
5	c.C857T^	GJA8	CTC>CTT	L268L	SYN	N/A	3.3%
6	c.T905C*	GJA8	TTG>TCG	L281C	NS	−3.97/0.00	3.3%

(Abbrevations: *Novel variations, ^Reported- ensembl, SYN-synonymous, NS-Non-synonymous, N/A-Not applicable).

### Summary of mutations in α-crystallin genes

The α-crystallin gene family consists of two similar genes coding for αA-crystallin (*CRYAA* located on chromosome 21q22.3) and αB-crystallin (*CRYAB* on chromosome 11q22.1) sharing 57% sequence identity. *CRYAB* contains 3 exons which encodes a 175 amino acid protein. Direct sequencing of the coding regions and of the flanking intronic sequences of *CRYAB* revealed one nucleotide change (rs11603779T>G) in the intronic region between exon 2 and 3 of *CRYAB*. The variation was found in 13.33% (4/30) case of congenital cataract. No nucleotide changes were found in controls. Improved splice site prediction for rs11603779T>G showed that this location is not present at splice site and may not create a splicing error in *CRYAB.*

### Summary of mutations in γ-crystallin genes

**Figure 2:**
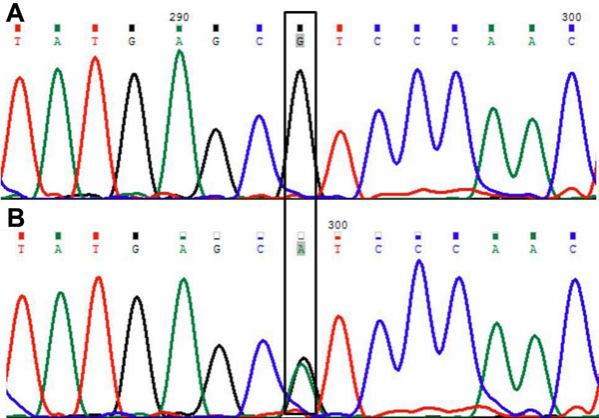
*CRYGC* DNA sequence in an affected and an unaffected individual. **A**: DNA sequence electropherogram of an unaffected individual showing wild type G at position 181. **B**: DNA sequence electropherogram showing the heterozygous 181G>A substitution that replaces Arginine by Histidine at codon 48 in the affected individual. The position of mutated (G>A) and wild type nucleotide G in an affected and an unaffected individual is indicated in box.

**Figure 3:**
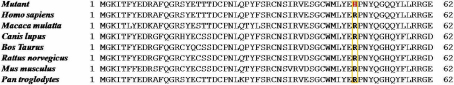
Multiple sequence alignment of the fourth Greek key motif of CRYGC is shown from *Homo sapiens* (codons 1–62), *Macaca mulatta, Canis lupus, Bos Taurus, Rattus norvegicus, Mus musculus*, and *Pan troglodytes*. The Arg48 residue is highly conserved.

The γ-crystallin gene family is mainly located in a cluster of six highly related genes (*CRYGA-CRYGF*) on human chromosome 2q33–35 and the seventh *CRYG* gene (*CRYGS*) on human chromosome 3. Mutations in *CRYGC* and *CRYGD* have been associated with congenital and hereditary cataract (Table 4). Direct sequencing of the coding region and of the flanking intronic sequences of *CRYGC* and *CRYGD* revealed three sequence variations. One heterozygous nucleotide change (c.G181A) was detected in exon 2 of *CRYGC*, resulting in the substitution of Arg to His at codon 48 (p.R48H; Figure 2) and was found in 13.33% (4/30) cases of congenital cataract. The multiple sequence alignments generated using FASTA3 (version 3 at the EBI) software showed that the Arg at position 48 of human *CRYGC* is highly conserved in *Macaca mulatta*, *Canis lupus*, *Bos taurus*, *Rattus norvegicus*, *Mus musculus*, and *Pan troglodytes* (Figure 3). Nucleotide change p.R48H was found to be non-pathogenic on insilico analysis (PANTHER and SIFT; Table 3). However as this change was in a highly conserved domain it may adversely affect protein function. None of the nucleotide changes were detected in control group. Two nucleotide changes in *CRYGD*; c.A313G in exon 3, resulting in synonymous change (p.R95R; rs2305430) and c.T564C (rs2305429) in the 3′UTR region, were also observed in 28 and 23 patients, respectively.

**Table 4:** Summary of the mutations identified in *CRYGD*, *CRYGC*, and *GJA8* with different congenital cataract phenotypes belonging to different ethnic groups.

**Gene**	**Nucleotide**	**Amino acid**	**Phenotype**	**Ethnicity**	**Reference**
*CRYGD*	c.43C>T	p.Arg15Cys	Punctate cataract, juvenile progressive, Coralliform/nuclear	Caucasian, Chinese	[70]
*CRYGD*	c.43C>A	p.Arg15Ser	Coralliform	Chinese	[71]
*CRYGD*	c.70C>A	p.Pro24Thr	Lamellar, Cerulean Coral-shaped- coralliform, Flaky-silica-like nuclear cataract, Fasciculiform	Indian, Moroccan, Caucasian, Australian, Chinese, Saudi Arabian	[46,72]
*CRYGD*	c.109C>A	p.Arg37Ser	with protein crystallization, Nuclear golden crystal	Czech boy, Chinese	[73]
*CRYGD*	c.168C>G	p.Tyr56Stop	Nuclear	Brazilian	[74]
*CRYGD*	c.176G>A	p.Arg59His	Aculeiform	Macedonian	[44]
*CRYGD*	c.181G>T	p.Gly61Cys	Coralliform	Chinese	[75]
*CRYGD*	c.320A>C	p.Glu107Ala	Nuclear	Hispanic	[76]
*CRYGD*	c.403C>A	p.Tyr134Stop	No data	Danish	[69]
*CRYGD*	c.418C>T	p.Arg140Stop	Nuclear	Indian	[41]
*CRYGD*	c.470G>A	p.Trp157Stop	Nuclear	Indian	[46]
*CRYGD*	c.494delG	p.Gly165fs	Nuclear	Chinese	[72]
*CRYGD*	c.229C>A	p.Arg77Ser	Anterior polar, Coronary	Indian	[77]
*CRYGC*	c.13A>C	p.Thr5Pro	Coppock-like	----	[44]
*CRYGC*	c.123insGCGGC	p.Gly41delinsGlyfsX62	Zonular pulverulent	----	[45]
*CRYGC*	c.502C>T	p.Arg168Trp	Lamellar/nuclear	----	[46]
*CRYGC*	c.327C>A	p.Cys109X	Nuclear	Chinese	[47]
*CRYGC*	c. 181G>A	p.Arg48His	Zonular and nuclear cataract	North Indian	Present study
*GJA8*	c.68G>C	p.Arg23Thr	Progressive dense nuclear	Iranian	[54]
*GJA8*	c.92T>C	p.Ile31Thr	Nuclear cataract	Chinese	[55]
*GJA8*	c.131T>A	p.Val44Glu	Cataract and microcornea	Indian	[68]
*GJA8*	c.134G>C	p.Trp45Ser	Jellyfish-like cataract and microcornea	Indian	[56]
*GJA8*	c.139G>A	p.Asp47Asn	Nuclear pulverulent cataract	English	[57]
*GJA8*	c.139G>T	p.Asp47Tyr	Nuclear cataract	Chinese	[58]
*GJA8*	c.142G>A	p.Glu48Lys	Zonular nuclear pulverulent	Pakistani	[59]
*GJA8*	c.191T>G	p.Val64Gly	Nuclear cataract	Chinese	[60]
*GJA8*	c.235G>C	p.Val79Leu	Full moon like with Y-sutural opacities	Indian	[61]
*GJA8*	c.262C>T	p.Pro88Ser	Zonular pulverulent	English	[62]
*GJA8*	c.262C>A	p.Pro88Gln	“Balloon-like”cataract with Y-sutural opacities	English, Indian	[63]
*GJA8*	c.565C>T	p.Pro189Leu	Nuclear cataract and microcornea	Danish	[69]
*GJA8*	c.593G>A	p.Arg198Gln	Posterior subcapsular cataract and microcornea	Indian	[68]
*GJA8*	c.670insA	Fs	Total cataract and nystagmus	Indian	[64]
*GJA8*	c.741T>G	p.Ile247Met	Zonular pulverulent cataract	Russian	[65]
*GJA8*	ins776G	Fs	Triangular nuclear cataract	German	[66]
*GJA8*	c.827C>T	p.Ser276Phe	Pulverulent nuclear cataract	Chinese	[67]
*GJA8*	c. 905T>C	p.Leu281Cys	Zonular Cataract	North Indian	Present study

### Summary of mutations in the *GJA8* (Connexin-50) gene

**Figure 4:**
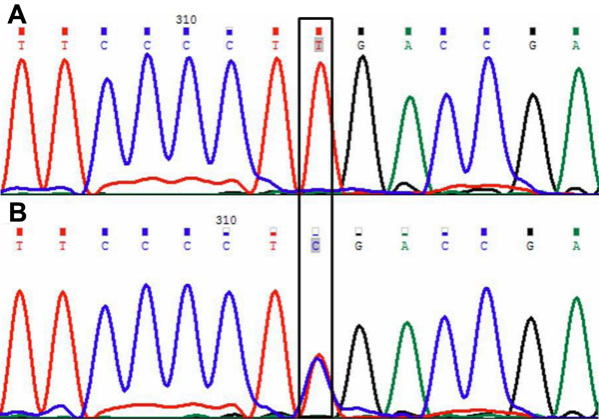
*GJA8* DNA sequence in an affected and an unaffected individual. **A**: DNA sequence electropherogram of an unaffected individual showing wild type T at position 905. **B**: DNA sequence electropherogram showing the heterozygous 905T>C substitution that replaces Leucine by Cysteine at codon 281 in the affected individual. The position of mutated (T>C) and wild type nucleotide G in an affected and an unaffected individual is indicated in the box.

**Figure 5:**
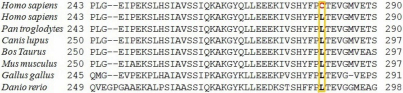
Multiple sequence alignment of the fourth Greek key motif of GJA8 is shown from *Homo sapiens* (codons 243–290), *Pan troglodytes, Canis lupus, Bos Taurus, Mus musculus*, *Gallus gallus* and *Danio rerio*. The Lys281 residue is highly conserved.

Direct sequencing of the amplified fragments of *GJA8* in congenital cataract patients identified two single base alterations (p.L268L and p.L281C). The change p.L268L was found in 3.33% (1/30) case of congenital cataract with anterior polar cataract whereas p.L281C (heterozygous) also found in 3.33% (1/30) cases. This case had lamellar/zonular form of cataract with nystagmoid movement. Both the nucleotide alterations (c.C857T and c.T905C) were in the second exon of *GJA8*. The nucleotide alteration c.T905C resulted in a novel amino acid substitution of leucine to cysteine at codon 281 (p.L281C; Figure 4) whereas the c.C857T nucleotide alteration leads to a synonymous amino acid substitution. GJA8 family protein sequences were obtained from NCBI website and multiple-sequence alignments of GJA8 family proteins from various species were obtained (Figure 5) using FASTA (version 3 at the EBI). This changed a phylogenetically conserved leucine to cysteine at codon 281 (p.L281C). Computational analysis (PANTHER and SIFT) of p.L281C predicted this nucleotide change as a pathogenic variant (Table 3). The remainder of the *GJA8* coding sequence showed no change. In addition, these nucleotide changes were not detected in 30 normal unrelated individuals from the same ethnic background.

### Comparative modeling study of γC-crystallin

**Figure 6:**
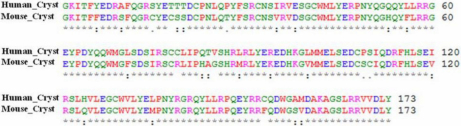
Sequence alignment of wild type human γC-crystallin and mouse γC-crystallin (PDB ID: 2V2U).

**Figure 7:**
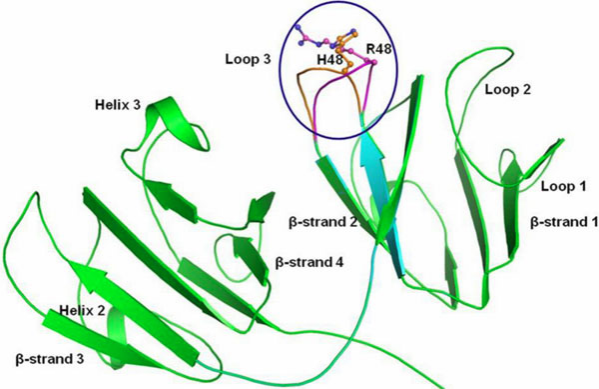
Change in conformation of loop3 in wild type (in magenta) and mutant (in orange) of human γC-crystallin.

Since the sequence identity between target (human γC-crystallin) and template (mouse γC-crystallin) was 84% (Figure 6), the structural reliability of the generated 3-dimensional homology model was high. The stability of the modeled wild-type and mutant (Arg48His) human γC-crystallin was checked by performing molecular dynamics simulation. These results indicated that the structures were stable. Both the wild type and mutant had very similar conformation with good geometry. The structure of wild type as well as mutant human γC-crystallin consisted of three small helices (each helix contains four residues), four anti-parallel β-strands and connecting loops (Figure 7).

### In-silico analysis

**Figure 8:**
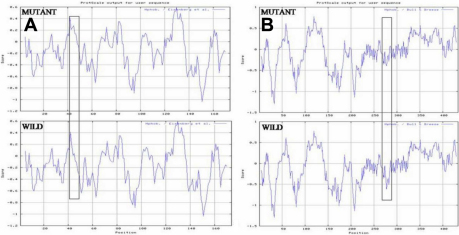
Hydrophobicity analysis. **A**: Comparision of hydrophobicity between wild type and mutant CRYGC. The protscale online software predict the effect of substitution on CRYGC protein hydrophobicity. Hydrophobicity of mutant protein increases around mutation point (R48H). **B**: Comparision of hydrophobicity between wild type and mutant GJA8. The protscale online software predict the effect of substitution on GJA8 protein hydrophobicity. Hydrophobicity of mutant protein increases around mutation point (L281C).

**Figure 9:**
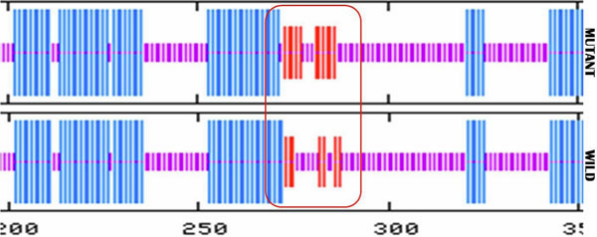
The predicted secondary structure of the mutant and wild type amino acid sequences. Target sequences are shown by red box which indicate that random coil has been replaced by the exented loop.

PANTHER and SIFT online tools were used for potential functional prediction of mutant proteins. After input the amino acid sequences of the wild-type CRYGC and GJA8 protein and their mutants, the PANTHER scores were −2.72 and −3.97, respectively, whereas the SIFT scores were 1.00 and 0.00, respectively, which meant that the variant (CRYGC:p.R48H) was predicted as “non-pathogenic” and the variant (GJA8:p.L281C) was predicted as “pathogenic” with high confidence. In comparison with the wild-type CRYGC and GJA8 protein, the hydrophobicity of the mutants CRYGC and GJA8 were dramatically increased (Figure 8). The secondary structure of mutant and wild type amino acid sequences of *GJA8* were analyzed by Antheprot 2000ver. 6.0 software (IBCP, Lyon,France) which showed that the mutation p.L281C lead to the replacement of random coil with extended loop (Figure 9). This replacement may be sufficient to change the secondary structure of the protein resulting in lens opacification.

### Genotype-phenotypes correlation

The genotype-phenotype correlation with the different morphological types of congenital cataract, their severity, visual acuity and different mutations have been tabulated (Table 5). The genotype and phenotype correlation coefficient (r value) between parameters like Degree of opacification, morphology of congenital cataract, visual acuity and mutations, showed no significance and hence no association between mutations and different parameters. Therefore no particular type of cataract was found to be associated with any particular mutant phenotype.

**Table 5:** Correlation coefficient and p value between crystallins and gap junction protein gene mutations and visual parameters (degree of opacification and visual acuity).

**Mutations**	**Degree of opacification**	**Visual acuity**
*CRYGC*:p.R48H	r=0.1903, p=0.3139	r=-0.0747, p=0.6945
*CRYGD*:p.R95R	r=0.0808, p=0.6711	r=-0.0490, p=0.7969
*CRYGD*:T564C	r=0.0955, p=0.6154	r=0.04662, p=0.8067
*CRYGD*:rs11603779T>G	r=0.0951, p=0.6170	r=0.0753, p=0.6922
*GJA8*:p.L268L	r=-0.2252, p=0.2315	r=-0.1110, p=0.5594
*GJA8*:p.L281C	r=-0.2252, p=0.2315	r=-0.2336, p=0.2394
Visual acuity	r=0.1915, p=0.3100	–

## Discussion

The transparency and high refractive index of the lens are achieved by the precise architecture of the fiber cells and the homeostasis of the lens proteins in terms of their concentration, stability, and supramolecular organization [40]. In this pilot study we identified six nucleotide variations (Table 3). Crystallin specific mutations (16.6%) were identified which is similar to the mutations detected in south Indian population [41]. We also detected 16.6% *GJA8* specific variations in congenital cataract cases. We identified two non-synonymous novel mutations, p.R48H(4/30) and p.L281C(1/30) in *CRYGC* and *GJA8*, respectively. Crystallins (α-, β-, and γ-crystallin) encode the major proportion of water soluble structural proteins of the lens fiber cells and are ubiquitous lens proteins. Functional changes and alteration of crystallin molecular properties could cause the breakdown of the lens microstructure and result in changes in the refractive index and increased light scattering.

### Description of mutations in *CRYGC* and associated phenotypes

It is reported that self aggregation or quaternary structural alteration of γ-crystallin is responsible for the phenotypic association with lens opacification as well as cataractogenesis [42,43]. To the best of our knowledge, four mutations in *CRYGC* have been reported in the literature (Table 4) [44-47]. The mutation p.R48H involves substitution of highly basic and polar charged Arginine with a neutral and less polar Histidine which may cause conformational changes. Arginine has well spread electron density enabling high solubility. It is a hydrophilic amino acid with a positive charge and lies within the extended strand on the surface of the molecule, interacting with water. Arginine is replaced by histidine, a hydrophobic amino acid compared to arginine. It has been reported that changing the solvation property of an amino acid residue on the surface of the γ-crystallin protein diminishes its solubility [48]. The distorted γC-crystallin may change its folding properties as shown in a study where the COOH-terminal domain folds before and nucleates the folding of the NH_2_-terminal domain in human γD-crystallin refolding [49]. The relatively loose or partially unfolded structure of mutant γC-crystallin may be susceptible to aggregation and insolubilization, which leads to cataract formation [50]. Another possible consequence of the R48H mutation may be related to the disturbances of the interactions between γC-crystallin and other crystallins [51].

**Figure 10:**
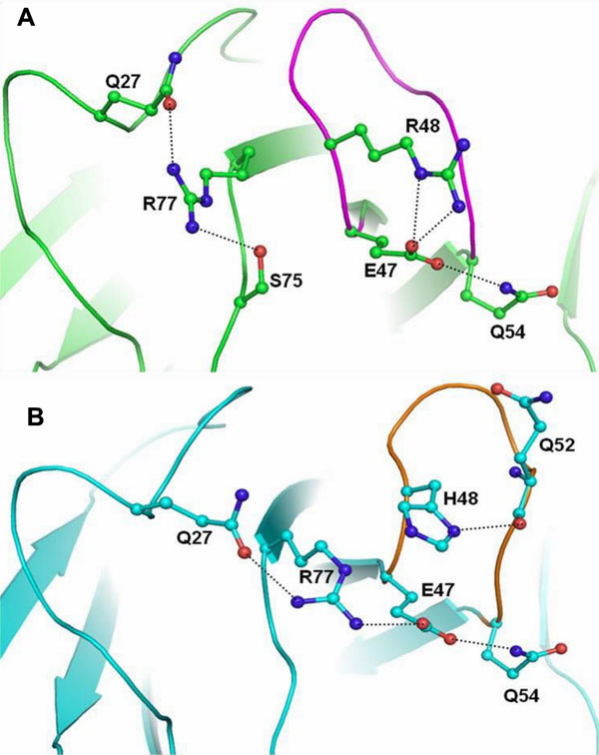
Hydrogen bond interactions. **A**: Conformation of loop3 (in magenta) in wild type human γC-crystallin. **B**: Conformation of loop3 (in orange) in mutant (R48H) human γC-crystallin.

The overall conformation, secondary structure elements and geometry of the conformers of wild-type and mutant human γC-crystallin were mainly similar. The most significant variation was observed in the conformation of the loop 3 region involving residues 47–54 which houses the mutation (Arg48His; Figure 7). The mutant has substitution of the longer and basic Arginine by a shorter Histidine possessing an imidazole ring. Thus this mutation alters the characteristic of this residue in both nature and length which is reflected in the difference in its interactions with neighboring amino acid residues The Arg48 in the wild type interacts with its adjacent acidic residue Glu47 which in turn forms a hydrogen bond with Gln54 thus stabilizing the loop 3 (Figure 10A). Thus the interaction of Glu47 with both residues Arg48 and Gln54 imparts an orientation to loop3. In the case of the mutant the shorter Histidine is no longer able to interact with Glu47 but instead interacts with Gln52 (Figure 10B). This loss of the stabilizing interaction enables Glu47 to adopt a different orientation and it in turn interacts with Arg77 while maintaining its interaction with Gln54. This alteration in the orientation of Glu47 and subsequently its interacting residues in the mutant, results in the modification of the relative orientation of the loop 3 comprising residues 47 to 54.

γ-Crystallins are long lived proteins of the lens and are generally characterized by both high stability and solubility. A key feature of γ-crystallins is that their surfaces are covered in ion pairs. The Arg head groups are most accessible to solvent water and therefore have a profound effect on the surrounding water, particularly important in lens. Since sequences of γ-crystallins are optimized for high solubility and minor changes to the surface can dramatically alter solution interaction properties. So the changes in protein conformations can decrease in solubility and stability of native protein which could result in aggregation of protein and thus opacification of lens. The R48H mutation interferes with the formation of two COOH-terminal Greek key motifs. Although the function of the Greek key motifs has not been elaborated in detail, computer-based analysis suggests that it may be responsible for particular protein–protein interactions in the lens, and it is postulated to be critical in the maintenance of lens transparency. The possible influence of the mutation on the structure as well as the function of γC-crystallin requires further investigation.

### Description of mutations in *GJA8* and associated phenotypes

*GJA8* is located on chromosome 1q21.1 and encodes a 50 kDa protein (connexin 50; Cx50). Cx-50 is a member of the connexin family of proteins that are important in the formation of gap junction channels [52] in human lens which are responsible for direct intercellular transfer of ions and molecules between adjacent cells [53]. Since the eye lens is an avascular structure, it relies heavily on an intercellular communication system constructed of gap junctions for preservation of tissue homeostasis and transparency [3,13]. Cx50 contains four transmembrane domains (M1, M2, M3, and M4) linked by two extracellular loops (E1 and E2), as well as an intracellular loop (CL), and intra-cytoplasmic NH_2_- and COOH-termini [54]. Mutant connexins are unable to participate in gap junction formation [13] and inhibit channel formation. To date, 22 mutations have been detected in *GJA8* in association with congenital cataract (Table 4) [54-69]. We identified a novel missense mutation, p.L281C, in GJA8 in cases with congenital cataract (zonular/lamellar cataract with nystagmus). Different mutations (Table 4) in the connexin gene are often associated pulverulent nuclear opacities [55,56,58,59,61,65,68,69]. However some studies [60,63,67] have detected mutations in *GJA8* which were associated with zonular/lamellar cataract phenotype as we found in this study. Insilico analysis of p.L281C mutation showed that the hydrophobicity of the mutant protein increased while the hydrophobic moment decreased (Figure 8). The predicted new characteristics of the mutant protein, which include altered interactions with other proteins, altered regulation activities of *GJA8*, and altered assemblies, may be the cause of the disease.

Our findings further expand the mutation spectrum of *GJA8* and *CRYGC* in congenital cataract. In summary, this study identified variations in 28 of 30 congenital cataract patients in north Indian population. Crystallin family (α- and γ-crystallin) accounts for 66% of the variations whereas connexins accounts for 44% of the total variations. It is notable that only two variations (*CRYGC*:p.R48H and *GJA8*:p.L281C) in *CRYGC* and *GJA8* detected in this study were predicted to be pathogenic which may cause congenital cataract. This study further confirms that *CRYGC* and *GJA8* play a major role in the maintenance of lens transparency and expands the mutation spectrum of both the genes in congenital cataract.
